# Pleiotropic Effect of a High Resolution Mapped Blood Pressure QTL on Tumorigenesis

**DOI:** 10.1371/journal.pone.0153519

**Published:** 2016-04-13

**Authors:** Xi Cheng, Harshal Waghulde, Blair Mell, Kathryn Smedlund, Guillermo Vazquez, Bina Joe

**Affiliations:** Program in Physiological Genomics, Center for Hypertension and Personalized Medicine, Department of Physiology and Pharmacology, University of Toledo College of Medicine and Life Sciences, Toledo, Ohio, United States of America; Max-Delbrück Center for Molecular Medicine (MDC), GERMANY

## Abstract

This study is focused on a translationally significant, genome-wide-association-study (GWAS) locus for cardiovascular disease (QT-interval) on human chromosome 17. We have previously validated and high resolution mapped the homologous genomic segment of this human locus to <42.5 kb on rat chromosome 10. This <42.5 kb segment in rats regulates both QT-interval and blood pressure and contains a single protein-coding gene, rififylin (*Rffl*). The expression of *Rffl* in the hearts and kidneys is differential between Dahl S and S.LEW congenic rats, which are the strains used for mapping this locus. Our previous study points to altered rate of endocytic recycling as the underlying mechanism, through which *Rffl* operates to control both QT-interval and blood pressure. Interestingly, *Rffl* also contributes to tumorigenesis by repressing caspases and tumor suppressor genes. Moreover, the expression of Methyl-CpG Binding Domain Protein 2 (*Mbd2*) in the hearts and kidneys is also higher in the S.LEW congenic strain than the background (control) Dahl S strain. *Mbd2* can repress methylated tumor suppressor genes. These data suggest that the S.LEW congenic strain could be more susceptible to tumorigenesis. To test this hypothesis, the S and S.LEW strains were compared for susceptibility to azoxymethane-induced colon tumors. The number of colon tumors was significantly higher in the S.LEW congenic strain compared with the S rat. Transcriptomic analysis confirmed that the chemical carcinogenesis pathway was significantly up-regulated in the congenic strain. These studies provide evidence for a GWAS-validated genomic segment on rat chromosome 10 as being important for the regulation of cardiovascular function and tumorigenesis.

## Introduction

This study is focused on a genome-wide-association-study (GWAS) locus for QT-intervals on human chromosome 17 [1]. In our previous study, we have already validated and high resolution mapped the homologous genomic segment of this human locus to <42.5 kb on rat chromosome 10. This was done by generating and characterizing S.LEW congenic rats with chromosomal segments from the normotensive Lewis (LEW) rat introgressed onto the genetic background of the hypertensive Dahl salt-sensitive (S) rat [2–7]. The locus in rats regulates both QT-interval and blood pressure and contains a single protein-coding gene, rififylin (*Rffl*) [7]. While there are no exonic variants, the expression of *Rffl* in the hearts and kidneys is differential between Dahl S and S.LEW congenic rats, which are the strains used for mapping this locus [7, 8]. Our study points to the altered rate of endocytic recycling as the underlying mechanism, through which *Rffl* operates to control both cardiac QT-intervals and blood pressure [7].

The functions of *Rffl* (rififylin) are: (a) rififylin is an endosome associated ubiquitin ligase; (b) rififylin is also known as caspases-8- and -10-associated RING protein 2 (*CARP-2*), which is an apoptotic inhibitor contributing to tumorigenesis by negatively regulating the levels of caspases (e.g., CASP8 and CASP10) and tumor suppressor genes through its direct ubiquitination and targeting for proteasomal degradation [9, 10]; (c) rififylin-mediated PKC activation is important for facilitating the migration of fibroblasts and tumor cells [11]. Interestingly, our previous results showed that the expression of a tumor regulatory gene, Methyl-CpG Binding Domain Protein 2 (*Mbd2*), in the hearts (Table 2 in Reference [7]) and kidneys (Table A1 in Reference [8]) was also expressed higher in the S.LEW congenic strain compared with the S rat. *Mbd2* can function as a transcription repressor by specifically binding to methylated promoters of tumor suppressor genes [12]. Dysregulation of *Mbd2* expression has also been reported in colorectal cancer [13]. These observations taken together with reports of hypertension to be associated with an increased risk of developing cancer [14, 15], were intriguing to construct the hypothesis that the congenic segment perturbs pathways relevant to tumorigenesis and renders the S.LEW congenic strain more susceptible to develop tumors.

Tumorigenesis was therefore examined in the S.LEW congenic strain compared with S. Data collected indicated that the congenic strain indeed demonstrated a higher susceptibility to develop azoxymethane-induced tumors. Cellular apoptosis was also markedly inhibited in the congenic strain. Colonic transcriptomic analysis indicated that the expected pathway of chemical carcinogenesis was upregulated in the congenic strain. Additionally, several pathways between the two strains were differential, including PPARα signaling and bile acid secretion pathways which were both upregulated, and the MAPKinase pathway which was downregulated in the congenic strain compared with S. This study therefore further extends the previous association of cardiovascular traits located on the chromosome 17 segment in humans and its homologous locus in rats, to the co-localization of the phenotype of tumorigenesis.

## Materials and Methods

### Reagents

Azoxymethane 13.4 M, ≥98% pure (AOM) and *In Situ* Cell Death Detection Kit, TMR red were purchased from Sigma (St. Louis, MO, USA).

### Animal experiments

All animal procedures and protocols described in this study were approved by the University of Toledo Institute Animal Care and Use Committee. Dahl salt-sensitive rats (SS/Jr) were from our colony and will be referred to as S. The congenic rat described in this study is the S.LEW(10)x12x2x3x5 (S.LEW congenic) strain [5]. Only male rats were used for the current study, in order to match the blood pressure QTL inference drawn from a previous study [7] conducted using male rats. All the experimental rats were weaned at 28–30 d of age and fed with a low salt (0.3% NaCl) Harlan Teklad diet. At six weeks of age, 14 S and 14 S.LEW congenic rats received intraperitoneal (IP) injection of AOM in sterile saline at a dose of 15mg/kg body weight once a week for two consecutive weeks, following which, two animals per cage, one S rat and one congenic rat, were housed in a room with a 12-hour light/dark cycle. Body weights were recorded weekly during the whole study period. At 30 weeks of age, all the rats were euthanized by carbon dioxide inhalation. Final body and organ weights for each rat were documented. Colon tissues were harvested, dissected longitudinally, and washed with saline. The number and sizes of the colon tumors were recorded by a researcher who was blinded to the identities of the rats.

### Tissue sectioning and in situ TUNEL assay

Areas of colon tissues without visible tumors were dissected from AOM-treated S and S.LEW congenic rats (n = 3/group). Tissues were embedded in O.C.T., frozen in the Peltier stage of the cryostat (Thermo Scientific R. Allan HM550 Cryostat). The embedded colon tissues were then processed for sectioning and tissue sections (10 μm) were collected onto Fisher Superfrost Plus-coated slides. Apoptosis assay of colon tissue sections was performed by using the *in situ* cell death detection kit (Roche, IN) according to the manufacturer’s protocol. Cell nuclei were stained blue with 4',6-diamidino-2-phenylindole (DAPI) and the apoptotic cells were stained red with the TUNEL reaction mixture containing the terminal transferase. In the negative control sections, the TUNEL reaction mixture was replaced with the Label Solution without the terminal transferase. Stained sections were scored as described in Smedlund KB, *et al* [16]. Briefly, three independent operators blinded to the study scored all the TUNEL stained sections with the following scoring system for apoptotic cells: none (score: 0), low (score: 1), intermediate (score: 2) or high (score: 3). The categorical scores were analyzed in the contingency table using GraphPad Prism version 6 and the p-value was computed using the chi-square test (χ^2^ test).

### RNA labeling and array hybridization

mRNA from colons of 3 S and 3 S.LEW congenic rats receiving AOM treatment were extracted for transcriptome analysis using TRIzol Reagent (Life Technologies) as per the manufacturer's protocol. RNA quantity and quality were measured by NanoDrop ND-1000. RNA integrity was assessed by standard denaturing agarose gel electrophoresis. The Rat 4x44K Gene Expression Array v3 representing 39,000+ rat genes and transcripts (Agilent) was used for the microarray experiments. Sample labeling and array hybridization were performed according to the Agilent One-Color Microarray-Based Gene Expression Analysis protocol (Agilent Technology) by Arraystar Inc. Briefly, total RNA from each sample was linearly amplified and labeled with Cy3-UTP. The Labeled cRNAs were purified by RNeasy Mini Kit (Qiagen). The concentration and specific activity of the labeled cRNAs (pmol Cy3/μg cRNA) were measured by NanoDrop ND-1000. 1 μg of each labeled cRNA was fragmented by adding 11 μl 10X Blocking Agent and 2.2 μl of 25X Fragmentation Buffer, then heated at 60°C for 30 min, and finally 55 μl 2X GE Hybridization buffer was added to dilute the labeled cRNA. 100μl of hybridization solution was dispensed into the gasket slide and assembled to the gene expression microarray slide. The slides were incubated for 17 hours at 65°C in an Agilent Hybridization Oven. The hybridized arrays were washed, fixed and scanned using the Agilent DNA Microarray Scanner (part number G2505C).

### Microarray analysis

Agilent Feature Extraction software (version 11.0.1.1) was used to analyze the acquired array images. Quantile normalization and subsequent data processing were performed with using the GeneSpring GX v12.1 software (Agilent Technologies). After quantile normalization of the raw data, gene expression detected in at least 3 out of 6 samples was chosen for further data analysis. Differentially expressed genes with statistical significance were identified through Fold Change. Hierarchical Clustering was performed using the Agilent GeneSpring GX software (version 12.1). Gene Ontology (GO) analysis and Kyoto Encyclopedia of Genes and Genomes (KEGG) pathway analysis were performed in the standard enrichment computation method using differentially expressed transcript data obtained with a cut-off value of 2-fold change.

The transcriptome data collected through the microarray study was deposited in NCBI's Gene Expression Omnibus (Cheng X and Joe B, 2015) and are accessible through GEO Series accession number GSE75280.

### cDNA analysis

mRNA from colon tissues of 6 S and 6 S.LEW congenic rats receiving AOM treatment were extracted using TRIzol Reagent (Life Technologies). cDNA was obtained through reverse transcription with SuperScript III (invitrogen). The cDNA obtained was PCR amplified using sense (5’ TCCCCTCAATCAGAACAAGG 3’) and antisense (5’ CGCTGAGGGTCTGACTTCAC 3’) primers designed to span the exon 3 and the exon 4 of the *Mbd2* transcript and the PCR product amplified was also confirmed by DNA sequencing. Transcript expression of *Mbd2* was analyzed by the real-time PCR (BioRad) and the expression levels relative to the housekeeping gene *Pgk1* were calculated by the 2^-ΔΔCT^ method.

### Statistical Analyses

Student’s t-test was used for all the statistical analyses except for in situ TUNEL assay. Data was presented as means ± SEMs. P < 0.05 was considered to be statistically significant. Chi-square test (χ^2^ test) was used for analyzing the categorical scores in the contingency table for TUNEL assay as described above.

## Results

### Number and size of colon tumors

**Fig 1:**
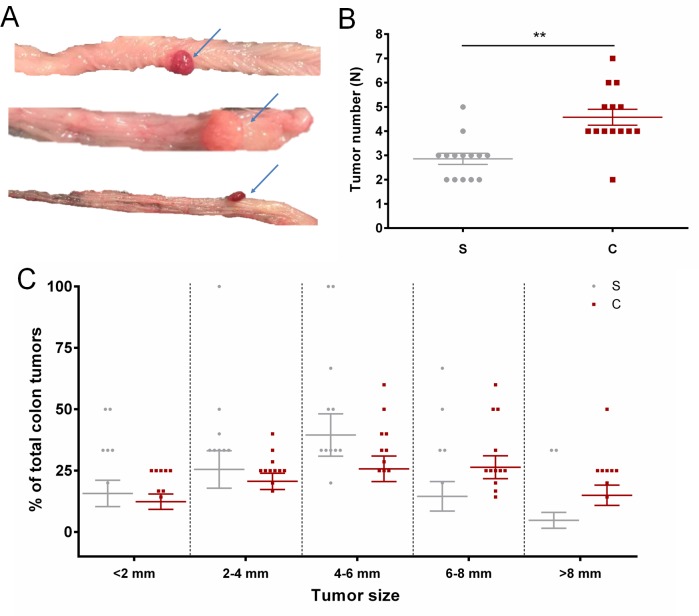
Colon tumors in the AOM-treated S and S.LEW congenic rats. (A) Blue arrows point to colon tumors of various sizes observed in experimental rats receiving AOM injection. (B) Scatter plot showing the distribution of the total number of colon tumors in all experimental rats. (C) Scatter plot comparing the percentage of colon tumors in different sizes between the S rats and the S.LEW congenic (C) rats. Each data point in the scatter plot represents one rat. The data points from rats that did not develop visible colon tumors are not included in the plot. All values were expressed as mean ± SEM. **: *P* < 0.01.

As shown in Fig 1A, colon tumors were successfully induced and observed in the experimental rats receiving the carcinogen AOM. The scatter plot in Fig 1B showed the distribution of the total number of colon tumors in each experimental rat and the plot indicated that the total number of colon tumors was significantly higher in the S.LEW congenic strain compared with the S rat (p = 0.0002). Colon tumors of different sizes were observed in both S and congenic rats. As shown in Fig 1C, the S rats had a higher percentage of relatively small colon tumors (size < = 6mm) and the S.LEW congenic rats had a higher percentage of relatively large colon tumors (size > 6 mm), which indicates a more rapid tumor progression in the S.LEW congenic strain compared with S. The results demonstrate that the congenic strain is genetically more susceptible to tumorigenesis.

### Apoptosis in colon tissues

**Fig 2:**
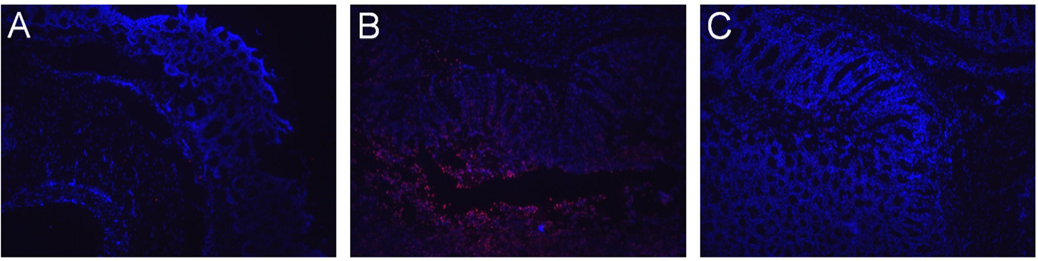
TUNEL assay of colon tissues from the AOM-treated S and S.LEW congenic rats (n = 3/group). All nuclei were stained with DAPI (blue). Cells with red stained nuclei were apoptotic cells. (A) Representative tissue section of the negative control, in which the TUNEL reaction mixture was replaced with the Label Solution without the terminal transferase (see Methods for details). (B) Representative tissue section from the S rat. (C) Representative tissue section from the S.LEW congenic strain. TUNEL staining was also scored by three independent operators blinded to the study groups.

Analysis of TUNEL stained colon sections from the S rats exhibited a more robust staining for apoptotic cells (p < 0.0001, χ^2^ test) compared to that from the S.LEW congenic strain. For example, 54% of all the sections showed TUNEL staining with weak and intermediate intensity in the S rats compared to 17% of all the sections in the S.LEW congenic rats. Representative images of TUNEL stained colon sections are provided in Fig 2 to visualize the enhanced red staining for apoptotic cells in S rats compared to the S.LEW congenic strain. These results demonstrated that the lower level of apoptosis in the colon sections of the S.LEW congenic strain is consistent with more colon tumors observed in the congenic strain.

### Differentially expressed genes in colon tissues

**Fig 3:**
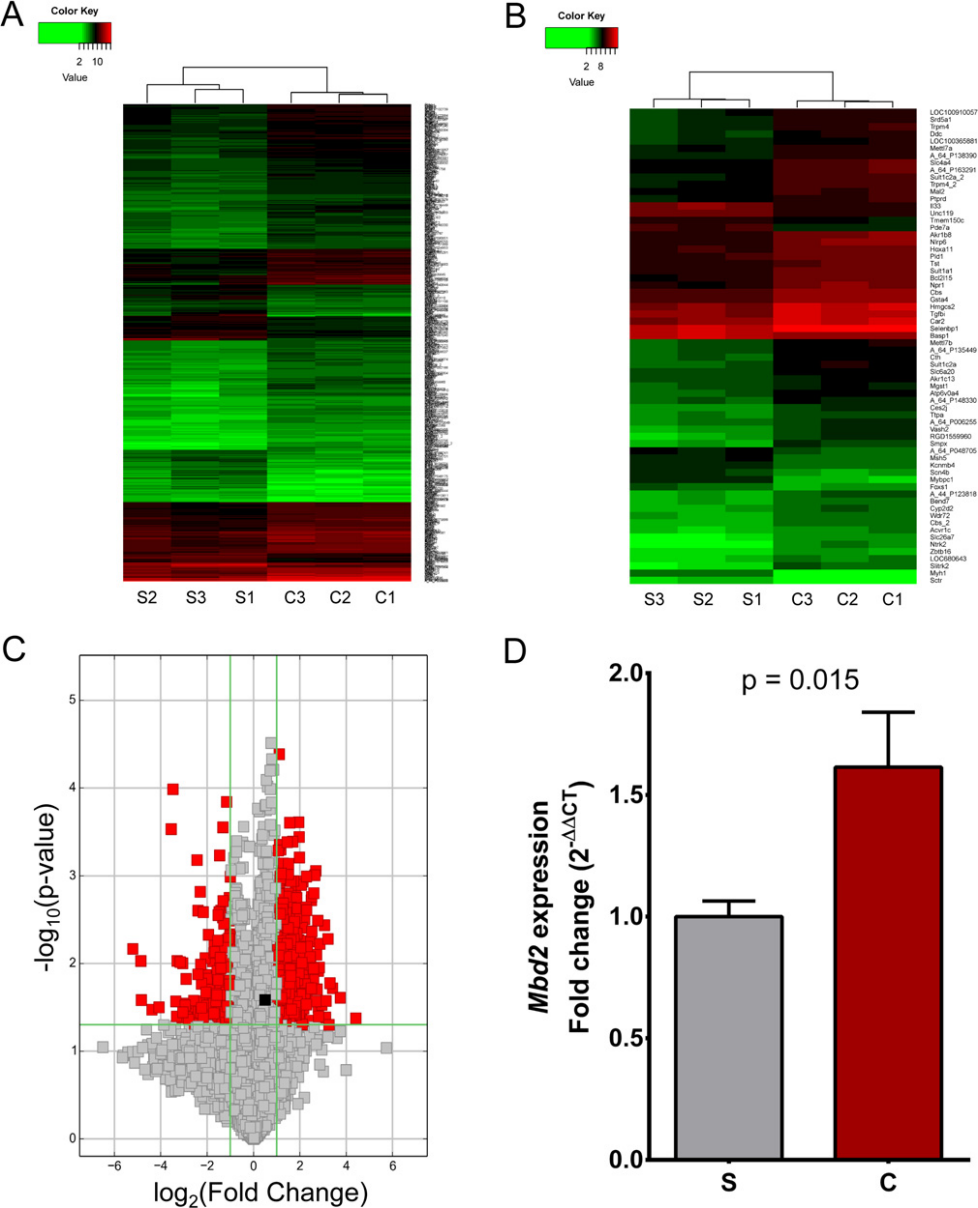
Gene expression profiles in the AOM-treated S and S.LEW congenic rats. (A) Heatmap of differentially expressed genes with a p-value cut-off 0.05 and a fold-change cut-off 2.0 between the S.LEW congenic (C) and S rats (n = 3/group). S1, S2, S3 are 3 individual S rats. C1, C2, C3 are 3 individual S.LEW congenic rats. Gene expression level is shown as a function of color, with lower expression in green and higher expression in red. (B) Heatmap of differentially expressed genes with a p-value cut-off 0.005 and a fold-change cut-off 2.5 between the S.LEW congenic (C) and S rats (n = 3/group). S1, S2, S3 are 3 individual S rats. C1, C2, C3 are 3 individual S.LEW congenic rats. Gene expression level is shown as a function of color, with lower expression in green and higher expression in red. (C) The volcano plot showing the significant up- and down-regulated genes in the S.LEW congenic strain compared with the S (n = 3/group). The horizontal green line represents a p-value with the cut-off 0.05. All the gray and red squares above this horizontal green line represent the differentially expressed genes with statistical significance (p < 0.05). The vertical green lines mark the limits for fold-change with the cut-off value of 2.0, whereby the red squares outside of the area between the two vertical green lines represent the genes with more than 2 fold-change and the gray squares between the two vertical green lines represent the genes with less than 2 fold-change. The black square denotes *Mbd2* with a 1.38 fold increased expression in the congenic strain. (D) Real-time PCR data confirming that colon *Mbd2* was expressed significantly higher in the S.LEW congenic (C) rats compared with the S rats (n = 6/group).

Transcriptome analysis indicated that a number of genes were differentially expressed between the colons of S and S.LEW congenic rats. Specifically, by using a p-value cut-off of 0.05 and a fold-change cut-off value of 2.0, 515 transcripts were upregulated and 178 transcripts were downregulated in the S.LEW congenic strain compared to S (S1 Table). A heatmap of all the differentially expressed transcripts is shown in Fig 3A. However, by using a more stringent p-value cut-off of 0.005 and a fold-change cut-off value of 2.5, the number of differentially expressed transcripts was further reduced to 53 transcripts upregulated and 13 transcripts downregulated in the S.LEW congenic strain compared to S (S2 Table). The heatmap of differentially expressed transcripts as analyzed using the more stringent criteria is shown in Fig 3B. Our previous transcriptome analysis showed that the expression of Methyl-CpG Binding Domain Protein 2 (*Mbd2*) in the hearts (Table 2 in Reference [7]) and kidneys (Table A1 in Reference [8]) was significantly higher in the congenic rats compared with the S rats. In concordance with this observation, the current microarray data showed that *Mbd2* in the colon was also significantly expressed higher in the congenic strain compared with the S (Fig 3C). This result was further confirmed by real-time PCR (p = 0.015) (Fig 3D).

### Functional and pathway enrichment analyses

**Fig 4:**
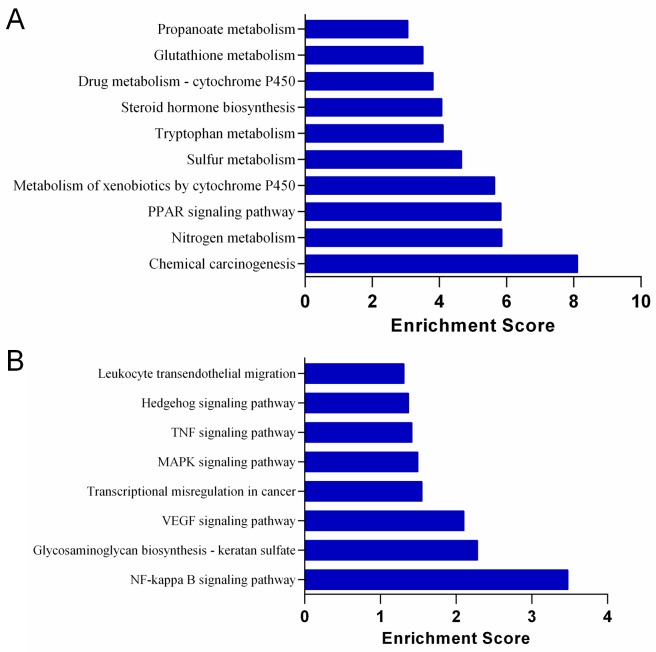
Kyoto Encyclopedia of Genes and Genomes (KEGG) pathways in the AOM-treated S and S.LEW congenic rats. The top significantly up-regulated (A) and down-regulated (B) KEGG pathways of differentially expressed genes in the S.LEW congenic rats compared with the S rats (n = 3/group). “Enrichment Score” = “-log_10_(Fisher p-value)” of the corresponding pathway in the microarray analysis.

**Fig 5:**
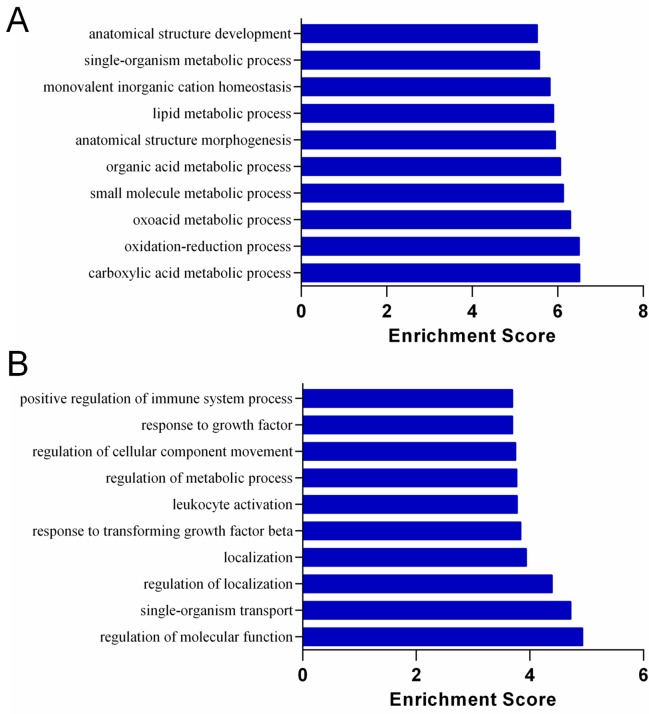
Gene Ontology for Biological Process (GO:BP) in the AOM-treated S and S.LEW congenic rats. The top significantly up-regulated (A) and down-regulated (B) GO:BP terms of differentially expressed genes in the S.LEW congenic rats compared with the S rats (n = 3/group). “Enrichment Score” = “-log_10_(Fisher p-value)” of the corresponding biological process in the microarray analysis.

**Fig 6:**
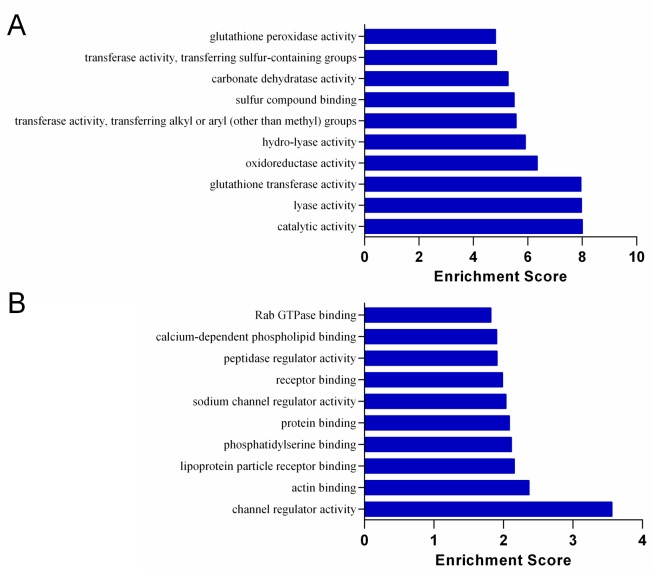
Gene Ontology for Molecular Function (GO:MF) in the AOM-treated S and S.LEW congenic rats. The top significantly up-regulated (A) and down-regulated (B) GO:MF terms of differentially expressed genes in the S.LEW congenic rats compared with the S rats (n = 3/group). “Enrichment Score” = “-log_10_(Fisher p-value)” of the corresponding molecular function in the microarray analysis.

Fig 4 showed the significantly up-regulated (Fig 4A) and down-regulated (Fig 4B) KEGG pathways in the S.LEW congenic rats compared with the S rats. Chemical carcinogenesis was the most significantly up-regulated pathway in the congenic rats compared with the S rats (Fig 4A), which was consistent with more colon tumors observed in the congenic strain receiving the chemical carcinogen AOM to induce colorectal carcinogenesis. The top significantly up-regulated and down-regulated biological processes (GO:BP) and molecular functions (GO:MF) in the S.LEW congenic rats compared with the S rats are shown in Figs 5 and 6, respectively.

## Discussion

The congenic rat models have been widely used as valuable genetic tools to explore the underlying mechanism of the origin and development of different diseases, including cancer and hypertension. For example, Schaffer BS, *et al* generated a panel of congenic rat strains to precisely map *Emca8*, a QTL located on rat chromosome 5, as genetic determinants of breast cancer susceptibility [17]. To identify blood pressure quantitative trait loci (BP QTLs) on the rat genome, our laboratory has generated several S.LEW congenic strains by transferring chromosomal segments from the normotensive Lewis (LEW) rat onto the genetic background of the hypertensive Dahl S rat. In one of the S.LEW congenic strains, which contains LEW alleles within the <42.5 kb BP QTL region, blood pressure was significantly increased and QT-intervals were significantly shorter compared with the S rat [7]. This BP QTL in rats contains only a single protein-coding gene, rififylin (*Rffl*). The expression of *Rffl* was significantly higher in the hearts and kidneys of the S.LEW congenic strain compared with S and the overexpression of *Rffl* is known to delay endocytic recycling, which has been functionally linked to hypertension [7, 8]. Intriguingly, *Rffl* plays an important role in the regulation of tumorigenesis by mediating tumor suppressor genes and regulating the tumor cell migration [9–11]. Further, the previous microarray study showed that Methyl-CpG Binding Domain Protein 2 (*Mbd2*), which functions as a transcription repressor of tumor suppressor genes, was expressed higher in the hearts (Table 2 in Reference [7]) and kidneys (Table A1 in Reference [8]) of the S.LEW congenic strain compared with S. The overexpression of these two tumor-related genes suggests that the S.LEW congenic strain could be more susceptible to tumorigenesis. After receiving the chemical carcinogen azoxymethane (AOM) to induce colorectal carcinogenesis, the number of colon tumors was significantly higher in the S.LEW congenic strain compared with S (Fig 1B). Using the colon tissues from these tumor rats, the microarray and real-time PCR results both showed that colon *Mbd2* was expressed higher in the S.LEW congenic strain compared with S (Fig 3). Consistently, the S.LEW congenic strain, which had more colon tumors, had lower apoptotic level in the colon (Fig 2). All of these results demonstrate the pleiotropic effects of this <42.5 kb genomic segment on both cardiovascular regulation and tumorigenesis.

Regarding the status of *Rffl* as a candidate gene, the evidence is weak. The microarray dataset did not reveal any differential expression of *Rffl* between the colons of S and S.LEW congenic strain, although evaluation through real-time PCR showed a higher expression of *Rffl* in the congenic strain compared with S. The data was however not statistically significant (data not shown). Therefore, it is possible that other variant/s within the congenic segment independently influence the expression of *Mbd2*. The studies above conclusively demonstrate that the S.LEW congenic strain is more genetically susceptible to tumorigenesis. To further explore the molecular mechanism of higher susceptibility to tumorigenesis, colonic transcriptome analysis was conducted. Based on the list of the differentially expressed genes between the S.LEW congenic strain and the S rat, the signaling pathways differentially regulated were obtained. Not surprisingly, chemical carcinogenesis, induced through the chemical carcinogen AOM, was the most significantly up-regulated pathway in the congenic rats compared with the S rats (Fig 4A). Interestingly, two other signaling pathways, peroxisome proliferators activated receptors (PPARs) signaling pathway (Fig 4A) and bile secretion (enrichment score: 2.89, data not shown), were also up-regulated in the congenic strain. PPARs have been demonstrated to promote tumor cell proliferation and angiogenesis, contributing to cancer development [18]. The transcriptome data demonstrated that the expression level of peroxisome proliferators activated receptor alpha (PPARα), one important component involved in the PPARs signaling pathway, was significantly higher in the congenic strain compared with S (S1 Table). A previous study reported that bile acids can induce PPARα expression and further contribute to colorectal carcinogenesis [19]. Therefore, one possibility is that upregulated secretion of bile acids in the congenic strain could further increase PPARα expression and thereby promote colorectal tumorigenesis.

Since the process of tumorigenesis is complex, it is likely that *Rffl* and *Mbd2* may not be the sole and direct candidate genes regulating tumorigenesis. The transcriptome analysis, which identified a large number of differentially expressed genes, indicates that there are many possibilities of multiple tumor-related signaling pathways being perturbed. The precise causative pathways remain to be sorted from the consequential pathways with further dissection of the congenic segment presented in the current study. For example, mitogen-activated protein kinases (MAPK) pathway was down-regulated in the congenic strain (Fig 4B), which is consistent with a previous study reporting that the down-regulation of MAPK pathway was observed in colorectal cancer [20]. Additionally, the tumor necrosis factor (TNF) pathway was also down-regulated in the congenic rats (Fig 4B). TNF plays important roles in diverse cellular events, such as apoptosis, necrosis, immune response, proliferation and angiogenesis, and it displays both pro- and anti-tumoral effects coupled with inflammatory mediation [21]. Overall, it is difficult to pinpoint any particular pathway independent of the other pathways, but the data is clear to point out that a short genomic segment could play multiple roles in both cardiovascular regulation and tumorigenesis.

In conclusion, this study has shown that a short genomic segment on rat chromosome 10, which was previously reported as a blood pressure quantitative trait locus and homologous to a GWAS-associated locus for QT-intervals on human chromosome 17, was also involved in tumorigenesis. Despite these important findings, our future perspective is to further investigate the identity of the quantitative trait nucleotides in the congenic segment that regulate the downstream physiological functions. We previously reported that there were 171 variants in this region, but none were exonic variants [7], which suggests that the variants from non-protein coding regions will be important to be considered as candidates for the observed pleiotropic effects.

## Supporting Information

S1 Table List of genes in the heatmap shown in Fig 3A. The list of genes was generated using a p-value cut-off of 0.05 and a fold-change cut-off value of 2.0 in the transcriptome analysis. In the ‘Direction’ column, ‘up’ indicates genes were upregulated in the S.LEW congenic strain compared to S and ‘down’ indicates genes were downregulated in the S.LEW congenic strain compared to S. P-value was calculated using unpaired t-test.
(DOCX)

S2 Table List of genes in the heatmap shown in Fig 3B. The list of genes was generated using a p-value cut-off of 0.005 and a fold-change cut-off value of 2.5 in the transcriptome analysis. In the ‘Direction’ column, ‘up’ indicates genes were upregulated in the S.LEW congenic strain compared to S and ‘down’ indicates genes were downregulated in the S.LEW congenic strain compared to S. P-value was calculated using unpaired t-test.
(DOCX)
