# Supplementary material for: Pleiotropic Effect of a High Resolution Mapped Blood Pressure QTL on Tumorigenesis
Source: PLoS One. 2016 Apr 13;11(4):e0153519. doi: 10.1371/journal.pone.0153519 (PMC4830557; doi:10.1371/journal.pone.0153519)
Supplement: S1 Table — The list of genes was generated using a p-value cut-off of 0.05 and a fold-change cut-off value of 2.0 in the transcriptome analysis. In the ‘Direction’ column, ‘up’ indicates genes were upregulated in the S.LEW congenic strain compared to S and ‘down’ indicates genes were downregulated in the S.LEW congenic strain compared to S. P-value was calculated using unpaired t-test. (DOCX) [file pone.0153519.s001.docx]

**S1 Table. List of genes in the heatmap shown in Fig 3A.**

| **Gene Symbol** | **Fold Change** | **Direction** | **P-value** |
| --- | --- | --- | --- |
| Speg | 2.7172136 | up | 0.011399936 |
| Il13ra1 | 2.0602589 | up | 0.026030833 |
| Cc2d2a | 2.1474591 | up | 0.001333813 |
| Thbd_2 | 2.1972672 | up | 0.007559121 |
| Mcee | 2.0534877 | up | 0.009478424 |
| Acot13 | 2.0905458 | up | 0.012321253 |
| Lmo4 | 2.0342834 | up | 0.016172173 |
| A_64_P102739 | 2.0653572 | up | 0.006986501 |
| Pvrl2 | 2.0477984 | up | 0.021123182 |
| Klk1c7 | 2.0736116 | up | 0.034463033 |
| Klk1 | 2.1682398 | up | 0.024963695 |
| Dhrs11 | 2.0058838 | up | 0.022581919 |
| Rassf6 | 2.366557 | up | 0.002676036 |
| Slc4a4 | 2.5883493 | up | 0.006983548 |
| Slc6a17 | 3.4332316 | up | 0.022320643 |
| Enpep | 3.3640679 | up | 0.017722491 |
| A_64_P127884 | 3.4926668 | up | 0.030566333 |
| Adamtsl1 | 2.7181126 | up | 0.047902511 |
| Car8 | 3.150101 | up | 0.022068499 |
| LOC501437 | 3.2693572 | up | 0.008072421 |
| A_64_P157820 | 2.9642854 | up | 0.025123594 |
| Acsf2 | 2.864001 | up | 0.023108333 |
| A_64_P307087 | 3.5079933 | up | 0.009345047 |
| Kcnh2 | 2.9080189 | up | 0.033190091 |
| Rasd2 | 2.153195 | up | 0.024670438 |
| Macrod1 | 2.0780898 | up | 0.028946174 |
| Akap1_2 | 2.0987389 | up | 0.03807702 |
| Chdh | 2.1266142 | up | 0.038236723 |
| A_64_P148693 | 2.0508571 | up | 0.040315822 |
| Cd99l2 | 2.0008791 | up | 0.011737863 |
| LOC685767_2 | 2.2076549 | up | 0.004800959 |
| A_64_P011240 | 2.423809 | up | 0.001722347 |
| Fam83h | 2.0403693 | up | 0.016577224 |
| Mettl7a | 2.6472125 | up | 0.003491691 |
| Tmem117 | 2.2310451 | up | 0.017125834 |
| Ugt2b10 | 2.4321617 | up | 0.029486201 |
| Cblc | 2.1622737 | up | 0.032178133 |
| A_64_P080384 | 2.0786416 | up | 0.010993513 |
| Car9 | 2.0110879 | up | 0.033763403 |
| Slc39a14 | 2.1209461 | up | 0.030831559 |
| Tmem139 | 2.2405144 | up | 0.019467383 |
| Cdhr1 | 2.4553257 | up | 0.014724229 |
| Rgs9 | 2.5961141 | up | 0.021137691 |
| Rimbp2 | 2.0718942 | up | 0.039517085 |
| Nat2 | 2.4385139 | up | 0.000722039 |
| Plet1 | 2.4175292 | up | 0.006984685 |
| Prelid2_2 | 2.7291832 | up | 0.005947096 |
| Fam3b | 2.4428157 | up | 0.002491532 |
| Sult1c2a | 4.8959732 | up | 0.002449709 |
| A_44_P515441 | 5.3068208 | up | 0.011172927 |
| Pzp | 6.9557729 | up | 0.04009268 |
| A_64_P138390 | 2.9540229 | up | 0.000247245 |
| Clybl | 2.5144799 | up | 0.009683317 |
| Ppp1r26_3 | 2.2103541 | up | 0.016964563 |
| Sstr2 | 2.1303726 | up | 0.013586788 |
| Rassf4 | 2.1044944 | up | 0.01167261 |
| Cfb | 2.3753183 | up | 0.00677278 |
| Bcam | 2.1368257 | up | 0.035211974 |
| Tmem98 | 2.1827551 | up | 0.040390686 |
| Zfp703 | 2.5580078 | up | 0.030026506 |
| Mettl24 | 2.1918064 | up | 0.023317108 |
| Fzd8 | 2.041903 | up | 0.022346853 |
| Rerg | 2.1102894 | up | 0.036679088 |
| Cd248 | 2.9223559 | up | 0.033065401 |
| Mpst | 3.0518583 | up | 0.005437778 |
| Trpm4 | 3.4169044 | up | 0.003376761 |
| LOC100910057 | 3.5903488 | up | 0.003013837 |
| Srd5a1_2 | 3.6959494 | up | 0.0023343 |
| Slc18a1 | 3.339232 | up | 0.036409115 |
| Tmem139_2 | 2.1003914 | up | 0.009605011 |
| Klk8 | 2.1875344 | up | 0.00900936 |
| RGD1565779 | 2.244283 | up | 0.022961632 |
| Sult1c2 | 2.9958052 | up | 0.01156639 |
| A_64_P076057 | 3.2115797 | up | 0.012314465 |
| Dnajb3 | 4.2158771 | up | 0.039359324 |
| Slc17a4 | 3.5041202 | up | 0.044916682 |
| Urad | 4.8799782 | up | 0.028287972 |
| Dnajb3_2 | 4.4823317 | up | 0.028405082 |
| LOC100365881 | 2.8378728 | up | 0.000727358 |
| Galnt7 | 2.376965 | up | 0.004921878 |
| Gal3st1 | 2.0775444 | up | 0.003029903 |
| Higd1a | 2.2791649 | up | 0.000945131 |
| A_64_P010333 | 2.3424147 | up | 0.007024032 |
| Ces2g | 2.0244773 | up | 0.021058672 |
| Coro2a | 2.1190278 | up | 0.016744058 |
| A_64_P008450 | 2.5266551 | up | 0.043368606 |
| Trim2_2 | 2.5082698 | up | 0.017558335 |
| Clcn2 | 2.8682186 | up | 0.014026881 |
| Slc44a3 | 2.855216 | up | 0.017676804 |
| Ddc_2 | 3.2587301 | up | 0.004377423 |
| Mvb12b | 2.1529747 | up | 0.017878795 |
| Cyp2j4_2 | 2.1827575 | up | 0.00586903 |
| Abhd14b | 2.3155401 | up | 0.028697113 |
| Mgll | 2.2594798 | up | 0.029270868 |
| RGD1311756 | 2.1324078 | up | 0.02296493 |
| Lama2 | 2.3671762 | up | 0.032339633 |
| Klk1c4 | 2.1595384 | up | 0.043294014 |
| Slc27a6 | 2.9741363 | up | 0.031906307 |
| Cyp4f40 | 2.6816605 | up | 0.008408243 |
| Acvr1c_2 | 2.2261265 | up | 0.001072945 |
| Slc6a20 | 2.7617967 | up | 0.000987646 |
| Nkx3-2 | 2.2544899 | up | 0.027140196 |
| Ctxn3 | 4.0974762 | up | 0.006561689 |
| Fbn2 | 3.0824628 | up | 0.017931001 |
| Lsamp | 4.2426565 | up | 0.009805939 |
| Igf2bp2 | 2.1333384 | up | 0.00810303 |
| Ambp | 2.2143943 | up | 0.038296947 |
| Foxa3 | 2.327671 | up | 0.025765462 |
| Sobp | 2.1949065 | up | 0.032015225 |
| Akap1 | 2.3086216 | up | 0.04632156 |
| Yes1 | 2.0663247 | up | 0.009439568 |
| Slc25a40 | 2.0304497 | up | 0.005631435 |
| Rab17 | 2.1582308 | up | 0.003891653 |
| Lyrm7 | 2.1642542 | up | 0.001122181 |
| Car12 | 2.7952398 | up | 0.039411508 |
| Alppl2 | 2.6447773 | up | 0.038546981 |
| Pfkfb4 | 2.3932515 | up | 0.012284218 |
| Slc18b1 | 2.1252509 | up | 0.021965809 |
| Cgn | 2.4162666 | up | 0.021627783 |
| Ces1f | 2.2139239 | up | 0.044931198 |
| Slc9a3r2 | 2.0194655 | up | 0.041640972 |
| Dcdc2_2 | 2.2937839 | up | 0.003094334 |
| Fam78b | 2.2607801 | up | 0.000498484 |
| Akr1c13 | 2.661693 | up | 0.002633706 |
| Artn | 2.4316961 | up | 0.003099275 |
| Cdkl1 | 2.3136362 | up | 0.003278194 |
| Zp2 | 2.7820621 | up | 0.022127437 |
| A4gnt | 2.8793889 | up | 0.010899232 |
| Tph1 | 2.579378 | up | 0.006576228 |
| Cd99l2_2 | 2.423702 | up | 0.002055702 |
| Prelid2 | 2.2196544 | up | 0.024086438 |
| LOC679641 | 2.019821 | up | 0.005916526 |
| A_64_P158118 | 2.2395672 | up | 0.009741782 |
| A_64_P158219 | 2.0476782 | up | 0.001395411 |
| Psd3 | 2.149217 | up | 0.003626337 |
| Amt | 2.0927135 | up | 0.002194906 |
| Ehhadh | 2.0628607 | up | 0.009311095 |
| Kat3 | 2.4652259 | up | 0.001605247 |
| A_64_P097979 | 2.2177701 | up | 0.001022507 |
| Slc35g1 | 2.8534769 | up | 0.021596487 |
| Rimbp2_2 | 2.4671688 | up | 0.038436173 |
| RGD1305807 | 2.1413207 | up | 0.020102552 |
| Mgst1 | 2.6523864 | up | 0.002802345 |
| Rbp7 | 2.0258879 | up | 0.00448039 |
| Lrrc19 | 2.3918937 | up | 0.037575537 |
| Mettl7b | 4.309276 | up | 0.003915394 |
| A_64_P135449 | 3.5336774 | up | 0.001889041 |
| Cth | 4.7996878 | up | 0.002283302 |
| Sult1c2a_2 | 5.6496838 | up | 0.001728873 |
| Spink4 | 4.474805 | up | 0.032384687 |
| Arnt2 | 4.4400373 | up | 0.014022286 |
| Hsd17b6 | 6.6600187 | up | 0.019763643 |
| Atp6v0a4 | 5.7090898 | up | 0.004299129 |
| Lcn2 | 4.2144357 | up | 0.008897536 |
| Gcg | 4.0151837 | up | 0.006998764 |
| Nox1 | 3.5766033 | up | 0.036325734 |
| Adamdec1_2 | 5.5225916 | up | 0.005557898 |
| A_64_P106533 | 2.1430535 | up | 0.038189495 |
| Cidea_2 | 2.2817902 | up | 0.036031498 |
| Rgs11 | 2.0057593 | up | 0.047321942 |
| Acacb | 2.0254623 | up | 0.021301081 |
| Hoxa2 | 2.1747324 | up | 0.032596465 |
| Fn3k | 2.4161639 | up | 0.023982161 |
| Wasf1 | 2.9779689 | up | 0.025783549 |
| PCOLCE2 | 2.4536821 | up | 0.034102694 |
| Sgca | 2.9509042 | up | 0.040009306 |
| Golt1a | 2.5856799 | up | 0.010266725 |
| Acot4 | 2.1176172 | up | 0.014063963 |
| Nags | 2.9191763 | up | 0.014643125 |
| Hpdl | 2.2783067 | up | 0.025032344 |
| Sema5a | 2.1282991 | up | 0.03383575 |
| Cldn8 | 3.5723861 | up | 0.018363319 |
| Gucy2c_2 | 3.0280209 | up | 0.036306756 |
| Trim36 | 2.2746421 | up | 0.025623879 |
| Coro6 | 2.5578236 | up | 0.018928077 |
| A_64_P148330 | 2.8094514 | up | 0.003940019 |
| Car7 | 2.2497606 | up | 0.018447332 |
| Ppp1r26 | 2.2364878 | up | 0.025664005 |
| Ghr | 2.1901139 | up | 0.006864986 |
| Epha4 | 2.3123031 | up | 0.045953445 |
| Bcmo1 | 2.4064612 | up | 0.009441438 |
| Kcnj12 | 2.7869895 | up | 0.025052812 |
| Gdnf | 3.4090403 | up | 0.029054141 |
| F13a1 | 3.3348137 | up | 0.00527318 |
| Fam129a | 2.9747567 | up | 0.010267414 |
| Rax | 3.1274265 | up | 0.018201375 |
| Gpr37 | 3.4671585 | up | 0.037940078 |
| LOC685767 | 4.3735018 | up | 0.005517519 |
| Hrc | 3.6538468 | up | 0.014185223 |
| Adra1d | 4.7694533 | up | 0.009044074 |
| Epha7 | 4.9860373 | up | 0.006437518 |
| Asic4 | 3.8758989 | up | 0.012913638 |
| Slitrk6 | 2.0922211 | up | 0.020972901 |
| Nav2 | 2.2021311 | up | 0.025003693 |
| A_64_P090851 | 2.1790421 | up | 0.020648164 |
| Chst15 | 2.1821523 | up | 0.006304501 |
| Gprc5c | 2.2751652 | up | 0.027148566 |
| Kif26b | 2.9548633 | up | 0.008425918 |
| Olr59 | 2.8954768 | up | 0.00739149 |
| A_64_P006255 | 3.2210609 | up | 0.0004076 |
| Tph1_2 | 3.3673741 | up | 0.005849451 |
| Kiaa0895l | 2.0799615 | up | 0.040503296 |
| Morn1 | 2.5349761 | up | 0.040611563 |
| A_64_P151889 | 2.005957 | up | 0.018917809 |
| Gspt2 | 2.2717065 | up | 0.015134014 |
| Rnf43 | 2.1136185 | up | 0.037026724 |
| Gna11 | 2.193501 | up | 0.014307074 |
| Ephb2 | 2.7704371 | up | 0.017317676 |
| A_64_P054953 | 2.4231013 | up | 0.028133384 |
| Gjb3 | 2.4233769 | up | 0.044091186 |
| Sdcbp2 | 2.199491 | up | 0.00225912 |
| Aamdc_2 | 2.1775754 | up | 0.00044056 |
| Aamdc | 2.4903474 | up | 0.000928756 |
| St6galnac2 | 2.2995719 | up | 0.01464745 |
| Slc4a4_2 | 3.4385306 | up | 0.001981503 |
| A_64_P163291 | 3.8559036 | up | 0.000244638 |
| Fabp2 | 3.216578 | up | 0.043010596 |
| Gsta3 | 2.5012704 | up | 0.007351736 |
| Etv5_2 | 2.7869855 | up | 0.006452674 |
| Aldh1b1 | 3.2697712 | up | 0.007623104 |
| Cyp2j4 | 2.3701491 | up | 0.033684169 |
| Emid1 | 2.3297255 | up | 0.017621787 |
| Slc12a8 | 2.5612626 | up | 0.009136046 |
| Ptprd_2 | 2.8267272 | up | 0.004285868 |
| Abo | 2.203629 | up | 0.003530463 |
| Trpm4_2 | 3.0208635 | up | 0.002350324 |
| Mal2 | 2.8534419 | up | 0.001703338 |
| Cst6 | 2.057517 | up | 0.03309949 |
| Cidea | 2.1102457 | up | 0.045987541 |
| Fam89a | 2.0672941 | up | 0.039513099 |
| Adamdec1 | 4.3500869 | up | 0.011611217 |
| Cfd | 2.9652053 | up | 0.030118297 |
| A_64_P003883 | 2.1186484 | up | 0.044255747 |
| Klk1c3 | 2.208377 | up | 0.035180135 |
| Fam195a_2 | 2.3159886 | up | 0.002315457 |
| Vipr2 | 2.0608988 | up | 0.026489502 |
| Me1 | 2.3825866 | up | 0.045099442 |
| Gstm2 | 2.7900784 | up | 0.01930662 |
| Pcca | 2.1316984 | up | 0.012608501 |
| Ppp1r14d | 2.1008566 | up | 0.015743297 |
| Clcn2_2 | 2.3675868 | up | 0.011153409 |
| Cluh | 2.2169215 | up | 0.046885841 |
| Akr1c14 | 3.3237818 | up | 0.032358931 |
| Nxph3 | 2.4181499 | up | 0.035823906 |
| Fat4 | 2.9504016 | up | 0.016708302 |
| Fst | 3.4011269 | up | 0.01647075 |
| Aspn | 4.6339512 | up | 0.02177454 |
| RGD1305645 | 3.5669289 | up | 0.039219517 |
| Akr1b8 | 3.8994572 | up | 0.000361021 |
| Nlrp6 | 3.9451231 | up | 0.000619269 |
| Dpep1 | 3.543705 | up | 0.006312004 |
| Pld1 | 2.672576 | up | 0.001720618 |
| Npr1 | 4.384657 | up | 0.001156695 |
| A_42_P686234 | 5.4231372 | up | 0.008442512 |
| Pparg | 3.0873499 | up | 0.011306224 |
| A_44_P260709 | 3.0954486 | up | 0.03338737 |
| Muc2 | 4.0103917 | up | 0.029452161 |
| Dmbt1 | 7.2563716 | up | 0.005966351 |
| Ptgr1 | 5.1035271 | up | 0.019013489 |
| Agtr1a | 5.8936928 | up | 0.011618564 |
| Cyp2c24 | 9.507592 | up | 0.049893604 |
| Fabp1 | 21.2157092 | up | 0.042143981 |
| A_44_P440944 | -3.005574 | down | 0.005808558 |
| Plscr2 | -3.2433032 | down | 0.005673385 |
| Dram1 | -2.3240176 | down | 0.019402292 |
| P2ry6 | -2.2333978 | down | 0.031655876 |
| Serpinb9 | -2.2798405 | down | 0.011057944 |
| Ppp1r18 | -2.0824374 | down | 0.009789608 |
| Akna | -2.5125004 | down | 0.049507029 |
| B3gnt2 | -2.5862403 | down | 0.048615788 |
| Tnfrsf25 | -2.0484174 | down | 0.032542127 |
| Ldlrad4 | -2.0089983 | down | 0.001025802 |
| A_64_P007283 | -2.0390858 | down | 0.018204826 |
| RGD1306227 | -2.1779862 | down | 0.017350601 |
| Arid5a | -2.7181649 | down | 0.039179615 |
| Tnnt3 | -2.6226994 | down | 0.024757644 |
| Ccl2 | -3.4164134 | down | 0.017568572 |
| Pcsk9 | -4.6386382 | down | 0.042456445 |
| B3gnt2_2 | -3.4504907 | down | 0.031025732 |
| Pthlh | -4.0675212 | down | 0.030116554 |
| Mefv | -4.7480938 | down | 0.030920208 |
| RGD1310110 | -5.2573492 | down | 0.013319887 |
| Rgs14 | -5.9970708 | down | 0.026356218 |
| Col17a1 | -8.4422499 | down | 0.041143455 |
| N4bp2l1 | -2.4848877 | down | 0.001932889 |
| Rasl11a | -2.0225181 | down | 0.004640808 |
| A_64_P151156 | -2.347486 | down | 0.012856742 |
| Kcnmb4 | -2.8045503 | down | 0.002598907 |
| Slc45a3 | -2.8260961 | down | 0.041010068 |
| Galnt14 | -2.8983245 | down | 0.02704233 |
| Msh5 | -5.2548032 | down | 0.002494389 |
| Wdr87 | -2.0347474 | down | 0.002684633 |
| A_64_P135984 | -2.2382335 | down | 0.000142926 |
| Rltpr | -2.4432217 | down | 0.032045043 |
| Nfatc2 | -2.044059 | down | 0.027928096 |
| A_64_P023793 | -2.2054027 | down | 0.030703186 |
| Havcr1 | -2.1462104 | down | 0.025971781 |
| Atp10d | -2.2873503 | down | 0.038311458 |
| A_64_P077236 | -2.1549445 | down | 0.049652933 |
| A_64_P048705 | -4.5084686 | down | 0.002586488 |
| Ehd3 | -3.2783893 | down | 0.007587096 |
| Rin3 | -2.8625476 | down | 0.025486437 |
| Chi3l1 | -5.8583023 | down | 0.04882454 |
| Ltbp2 | -5.2870857 | down | 0.033073572 |
| Stx11 | -7.7388311 | down | 0.029926412 |
| Aim2 | -20.8743707 | down | 0.033645912 |
| RGD1562865 | -16.9192643 | down | 0.031520027 |
| Ceacam10 | -29.0601813 | down | 0.009383731 |
| A_64_P102598 | -2.0283754 | down | 0.004870675 |
| Tmem150c | -2.5081301 | down | 0.000279244 |
| Susd3 | -2.7525123 | down | 0.011364648 |
| Fam69a | -2.5664978 | down | 0.027980735 |
| Anxa3 | -2.7072773 | down | 0.029771462 |
| Slc16a3 | -2.4006782 | down | 0.014088431 |
| Gadd45b | -2.6592422 | down | 0.011304372 |
| A_64_P014178 | -2.2593473 | down | 0.038809941 |
| Guca2b | -2.1334365 | down | 0.041152761 |
| Pde7a_2 | -3.8468 | down | 0.004706498 |
| LOC102557467 | -2.5244029 | down | 0.012477363 |
| A_44_P185294 | -2.3575668 | down | 0.006588778 |
| Cry1 | -2.2535081 | down | 0.043260135 |
| Celf2 | -2.2836383 | down | 0.021379448 |
| LOC102547811 | -2.03885 | down | 0.005919447 |
| Ppm1m | -2.0291413 | down | 0.015322194 |
| Sacs | -2.0542731 | down | 0.017293472 |
| A_64_P076436 | -2.1685509 | down | 0.009740935 |
| Col20a1 | -2.0781637 | down | 0.003219137 |
| A_64_P108459 | -2.1951099 | down | 0.013287477 |
| Pwp2 | -2.3315556 | down | 0.035592959 |
| Fancb | -2.1673498 | down | 0.010523559 |
| Slc45a3_2 | -2.709428 | down | 0.01466382 |
| A_44_P760360 | -2.0011075 | down | 0.048225079 |
| Traf1 | -2.3390543 | down | 0.018963665 |
| Micall1 | -2.2377761 | down | 0.039003536 |
| Gpnmb | -2.4353138 | down | 0.003494495 |
| A_64_P087040 | -2.1076094 | down | 0.005203293 |
| Stap1 | -2.0460201 | down | 0.015748813 |
| Pxdc1 | -3.8244837 | down | 0.014921687 |
| Bcl6 | -4.7322775 | down | 0.016994176 |
| Prkcb | -4.1422384 | down | 0.013517264 |
| B3gnt7 | -28.6846631 | down | 0.026129308 |
| Slc37a2 | -10.0029043 | down | 0.039984652 |
| Fam55b | -8.9650165 | down | 0.009624589 |
| A_64_P133104 | 2.1022585 | up | 0.029155339 |
| A_64_P035945 | 2.0267168 | up | 0.012449604 |
| Sgpp2 | 2.0862654 | up | 0.021384899 |
| Duxbl1 | 2.1152356 | up | 0.039862028 |
| Nav2_2 | 2.1050323 | up | 0.013107556 |
| Ankrd34a | 2.317968 | up | 0.023855744 |
| Rasgrf2 | 2.5816414 | up | 0.021143623 |
| A_64_P082171 | 2.1391048 | up | 0.043326194 |
| Spdya | 2.0149311 | up | 0.045388219 |
| Hmcn2_4 | 2.814441 | up | 0.018145748 |
| A_64_P021892 | 2.6211942 | up | 0.0157464 |
| Mob3b | 2.6620836 | up | 0.011525399 |
| Tle6_2 | 2.1999146 | up | 0.004560362 |
| Map3k15 | 2.1666599 | up | 0.008131292 |
| Als2cl | 2.2126746 | up | 0.020998248 |
| Slc25a24 | 2.0541509 | up | 0.010987437 |
| A_64_P138537 | 2.1159729 | up | 0.005992682 |
| Ppara | 2.261238 | up | 0.023071038 |
| Arhgap20 | 2.6474822 | up | 0.019682827 |
| Gpr149 | 2.1142671 | up | 0.048171913 |
| Lppr3 | 2.8942408 | up | 0.021788568 |
| Insc | 2.5884966 | up | 0.035343473 |
| Lmo1 | 2.4500322 | up | 0.005798646 |
| Tle6 | 2.5081467 | up | 0.006730522 |
| Ifitm6 | 2.3498889 | up | 0.003646585 |
| Ttc21b | 2.081611 | up | 0.039563093 |
| Sv2b | 2.1821929 | up | 0.047839742 |
| A_64_P143674 | 2.1185653 | up | 0.032540606 |
| Cd163 | 3.6133092 | up | 0.038148275 |
| LOC365985 | 4.0536414 | up | 0.022508038 |
| Kctd8 | 3.1077376 | up | 0.026932729 |
| Ttpa | 3.0529996 | up | 0.001684656 |
| Capsl | 2.8881328 | up | 0.012839852 |
| Sytl4 | 2.2947826 | up | 0.034207656 |
| Cndp1 | 2.2855738 | up | 0.008975604 |
| Ppp1r26_2 | 2.8998949 | up | 0.023699283 |
| Sstr2_2 | 3.333275 | up | 0.009420777 |
| Col23a1 | 2.5166341 | up | 0.013997993 |
| Tmtc1 | 2.5555567 | up | 0.018555267 |
| A_64_P082166 | 2.3136322 | up | 0.033110362 |
| Abcd2 | 2.4411369 | up | 0.034871193 |
| Evc | 2.3181494 | up | 0.049034424 |
| Nr3c2 | 2.1918016 | up | 0.039295167 |
| Etv5 | 2.125783 | up | 0.012378927 |
| Csrnp3 | 2.2067417 | up | 0.003215981 |
| Cgref1 | 2.6277655 | up | 0.006035561 |
| Abcb9 | 2.5675584 | up | 0.022910738 |
| A_64_P150304 | 3.0539707 | up | 0.03070994 |
| Prelid2_3 | 2.1263831 | up | 0.001603719 |
| LOC691046 | 2.0931432 | up | 0.000512662 |
| Palmd | 2.265122 | up | 0.005890391 |
| L3hypdh | 2.0468163 | up | 0.021820974 |
| Hook1 | 2.0813528 | up | 0.016863285 |
| Sytl4_2 | 2.3352243 | up | 0.017519878 |
| Angpt1 | 2.6053901 | up | 0.016344337 |
| Ghrl | 5.0394191 | up | 0.011903883 |
| Ces2c | 3.9995114 | up | 0.009202427 |
| Cftr | 3.1828436 | up | 0.020803566 |
| Ces2j | 4.1681466 | up | 0.001263659 |
| Vash2 | 6.4438754 | up | 0.000877682 |
| RGD1559960 | 6.830004 | up | 0.003236271 |
| Cyp4b1 | 4.8834575 | up | 0.023839995 |
| Brinp3 | 6.9367073 | up | 0.012167898 |
| Smpx | 5.6342002 | up | 0.001891257 |
| Myoc | 6.9121314 | up | 0.031738706 |
| Colec10 | 6.4266429 | up | 0.027051965 |
| Tshr | 5.63074 | up | 0.019193669 |
| C6 | 5.4831648 | up | 0.009268971 |
| Myom2 | 9.8406317 | up | 0.016754826 |
| Pde11a | 7.6997124 | up | 0.029558899 |
| RGD1305928 | 8.6122347 | up | 0.041200326 |
| Pax4 | 2.6908586 | up | 0.018004342 |
| LOC100911410 | 2.8820646 | up | 0.005511905 |
| Lmx1a | 3.0034795 | up | 0.008248064 |
| Srd5a1 | 3.0071892 | up | 0.022274106 |
| Slc38a4 | 3.7364491 | up | 0.015406404 |
| Ugt8 | 6.0119823 | up | 0.011432361 |
| Cldn15 | 4.6606251 | up | 0.017176781 |
| A_64_P120986 | 8.735594 | up | 0.026673264 |
| Hsd3b5 | 13.3569438 | up | 0.024563503 |
| Fut9 | 5.4520884 | up | 0.044855126 |
| RGD1565374 | 4.8342111 | up | 0.029275443 |
| Cryba2 | 2.1822951 | up | 0.024247247 |
| A_64_P118225 | 2.1031748 | up | 0.024563177 |
| A_64_P007812 | 2.0055226 | up | 0.031767741 |
| Nrp2 | 2.1956026 | up | 0.036805664 |
| Dcdc2 | 2.6030192 | up | 0.010962231 |
| Arhgef38 | 2.0192917 | up | 0.01254288 |
| Psrc1 | 2.0493351 | up | 0.014262677 |
| LOC100912849 | 2.0298916 | up | 0.005577233 |
| Cbs_2 | 2.7287104 | up | 0.000412964 |
| A_64_P051070 | 2.2187095 | up | 0.002705044 |
| RGD1307461 | 2.0902999 | up | 0.001343973 |
| LOC100362814 | 2.1051917 | up | 0.023990522 |
| Mrc1 | 2.0370305 | up | 0.043831409 |
| Slc16a14 | 2.3781032 | up | 0.031404422 |
| A_64_P010714 | 2.2829857 | up | 0.003168684 |
| Plch1 | 2.618838 | up | 0.037336424 |
| Prlr | 3.5294647 | up | 0.006419375 |
| Cyp2d2 | 3.6525786 | up | 0.004563305 |
| Wdr72 | 2.7887728 | up | 0.001579095 |
| A_44_P123818 | 4.8627991 | up | 0.001016516 |
| Syne1 | 2.4541663 | up | 0.032556204 |
| Gtf2a1l | 2.7372165 | up | 0.017437717 |
| A_64_P127888 | 3.1245366 | up | 0.030643476 |
| Bend7 | 3.065226 | up | 0.00115393 |
| Frem3 | 3.2366188 | up | 0.020035709 |
| A_64_P052872 | 3.1937779 | up | 0.037861598 |
| Slitrk5 | 4.1132186 | up | 0.045840026 |
| Lrrc10b | 2.2895211 | up | 0.035760918 |
| A_64_P060296 | 2.9436432 | up | 0.032377073 |
| Oosp1 | 3.8500603 | up | 0.005410504 |
| LOC100911661 | 3.8767724 | up | 0.012894142 |
| Dlx1 | 2.8888654 | up | 0.03449862 |
| Lgi1 | 4.2815187 | up | 0.04952672 |
| Xpnpep2 | 3.3690286 | up | 0.007790395 |
| LOC680643 | 4.605212 | up | 0.003122024 |
| Tff2 | 3.5721291 | up | 0.037410691 |
| Dmrtc1a | 3.0937277 | up | 0.041879451 |
| A_44_P1070649 | 3.2131617 | up | 0.026977501 |
| Esm1 | 2.5884918 | up | 0.04170529 |
| Ephx2 | 2.274484 | up | 0.016239129 |
| Neurod1 | 2.0560943 | up | 0.017103649 |
| RGD1562080 | 2.032646 | up | 0.0310107 |
| Col13a1 | 2.0542914 | up | 0.008738054 |
| Aknad1 | 2.0463912 | up | 0.033127353 |
| Prss46 | 2.2565296 | up | 0.003209513 |
| Efhc2 | 2.3826733 | up | 0.00777131 |
| Acvr1c | 2.6530769 | up | 0.001881247 |
| A_64_P047455 | 2.4975109 | up | 0.015092774 |
| Shroom4 | 2.205349 | up | 0.02160342 |
| A_64_P047037 | 2.0441538 | up | 0.028401387 |
| LOC102551931 | 2.309085 | up | 0.027457447 |
| Lca5l | 2.3000434 | up | 0.031004167 |
| Wnt7a | 2.1519727 | up | 0.047232497 |
| A_64_P110789 | 2.1186608 | up | 0.032185023 |
| Cyp2c23 | 2.0032737 | up | 0.030320018 |
| Megf9 | 2.8567539 | up | 0.015252471 |
| Slc44a1 | 2.7230918 | up | 0.046588811 |
| Adamtsl1_2 | 2.2850901 | up | 0.023789471 |
| Thbd | 2.5388164 | up | 0.019312635 |
| Zbtb16 | 3.0732895 | up | 0.002284915 |
| Chrna10 | 2.3128045 | up | 0.02459761 |
| Sdr42e1 | 2.2305647 | up | 0.014958777 |
| Pde4c | 2.0263452 | up | 0.032288013 |
| A_64_P053756 | 2.1995043 | up | 0.020461061 |
| Slc12a1 | 2.1166677 | up | 0.002880764 |
| Gpr39 | 3.2704444 | up | 0.006585091 |
| Cml5 | 7.1437079 | up | 0.011136833 |
| LOC100361092_2 | 6.0111105 | up | 0.015017014 |
| Slc26a7 | 6.2250788 | up | 0.000982739 |
| Ntrk2 | 6.0686059 | up | 0.00157409 |
| LOC102548544 | 4.3153482 | up | 0.008376492 |
| LOC100361092 | 4.7046 | up | 0.022584039 |
| A_44_P809486 | 3.8961683 | up | 0.0389388 |
| Slitrk2 | 8.3412911 | up | 0.003657215 |
| Arx | 6.5660793 | up | 0.007950073 |
| Inmt | 4.5887024 | up | 0.013676402 |
| Tnfaip8l3 | 3.693138 | up | 0.013701952 |
| Abcb4 | -2.0622203 | down | 0.047063146 |
| Vom1r73 | -2.161343 | down | 0.024878668 |
| Hrh3 | -2.2867492 | down | 0.042907772 |
| Rp1 | -3.2694951 | down | 0.037048059 |
| A_64_P166241 | -2.9121736 | down | 0.039351907 |
| Kcnk13 | -2.939037 | down | 0.016991136 |
| LOC501315 | -2.8216843 | down | 0.012679711 |
| Bcl6b | -2.274919 | down | 0.044626835 |
| Gal3st2 | -2.9151573 | down | 0.005458177 |
| A_64_P164751 | -2.2135208 | down | 0.00537033 |
| Anxa13 | -3.9561218 | down | 0.046352295 |
| Scn4b | -5.4382765 | down | 0.00066085 |
| Serpine1 | -4.0868032 | down | 0.029836094 |
| LOC683963 | -4.8651043 | down | 0.010267049 |
| Egr2 | -7.10952 | down | 0.030451015 |
| Ccdc113 | -5.1792195 | down | 0.031828682 |
| Mybpc1 | -11.7554536 | down | 0.000293965 |
| Zfp217 | -2.2546085 | down | 0.036650935 |
| Skil | -2.4597286 | down | 0.049458261 |
| Dsg1 | -2.2095648 | down | 0.031901245 |
| A_64_P138221 | -2.9706678 | down | 0.012256471 |
| Foxs1 | -2.5156862 | down | 0.002983204 |
| A_64_P111089 | -2.00067 | down | 0.034383802 |
| Pde7a | -3.3041838 | down | 0.02869101 |
| A_64_P072170 | -3.0281089 | down | 0.022000784 |
| A_64_P031134 | -3.1503413 | down | 0.007387288 |
| A_64_P048416 | -2.3464374 | down | 0.033225031 |
| Celsr1 | -2.7428423 | down | 0.030089269 |
| LOC24906 | -2.0244233 | down | 0.002138493 |
| Gpr65 | -5.6912742 | down | 0.04015898 |
| Sphk1 | -7.5253172 | down | 0.015033453 |
| Htra4 | -6.9615798 | down | 0.043528302 |
| Bcl2a1 | -10.2393167 | down | 0.026922785 |
| Rassf5 | -8.2593535 | down | 0.03602844 |
| Cox8b | -5.2189663 | down | 0.035482801 |
| Tcrb | -4.3182577 | down | 0.049269197 |
| Mybph | -4.7311959 | down | 0.031895966 |
| Gp2 | -6.0574685 | down | 0.043900595 |
| A_64_P046175 | -2.9946455 | down | 0.022834772 |
| Spo11 | -3.409549 | down | 0.027605869 |
| Ttn | -4.4911017 | down | 0.040818114 |
| Retnlb | -36.9939975 | down | 0.006814515 |
| Lrp2 | -4.8683591 | down | 0.03614165 |
| LOC100359977 | -4.3577479 | down | 0.048901728 |
| A_64_P133484 | -3.4915684 | down | 0.049117303 |
| Gpr84 | -4.5546509 | down | 0.039065028 |
| Fer1l4 | -7.5108957 | down | 0.028784575 |
| RGD1566226 | -5.0229429 | down | 0.026810047 |
| A_64_P053312 | -7.4192912 | down | 0.033481612 |
| Cd52 | -2.7140611 | down | 0.022844678 |
| A_64_P003433 | -2.9609336 | down | 0.01519138 |
| A_64_P035996 | -2.7729954 | down | 0.037366354 |
| Spns3 | -3.8750861 | down | 0.025254803 |
| Slc15a1 | -9.849904 | down | 0.009329532 |
| Themis | -8.3099562 | down | 0.009973626 |
| Myh1 | -11.1713585 | down | 0.000102875 |
| LOC689230 | -5.140693 | down | 0.046249009 |
| Cela1 | -4.2592434 | down | 0.00866593 |
| Ppil6 | -2.6062501 | down | 0.03016382 |
| A_64_P068444 | -2.5756435 | down | 0.045899797 |
| Cacna2d3 | -3.7476078 | down | 0.016984172 |
| Ccdc155 | -3.2638846 | down | 0.049843597 |
| Slc30a2 | -2.9364473 | down | 0.031698284 |
| Wnt10a | -2.7502379 | down | 0.019033237 |
| Onecut1 | -2.5560439 | down | 0.047055754 |
| Map3k5 | -2.1199346 | down | 0.028847364 |
| LOC100910611 | -2.7052023 | down | 0.008541233 |
| Jmjd4 | -2.0283293 | down | 0.03501982 |
| Ms4a2 | -2.6632603 | down | 0.008046761 |
| Olr851 | -3.076234 | down | 0.026882939 |
| Camta1 | -2.7290166 | down | 0.020396439 |
| A_64_P030479 | -2.4450734 | down | 0.021766134 |
| LOC102556209 | -2.0410142 | down | 0.013267741 |
| Sctr | -2.8950302 | down | 0.00278897 |
| Spink3 | -4.1627685 | down | 0.021360222 |
| Olr1637 | -3.0111711 | down | 0.032700591 |
| Kcnh2_2 | 2.2617365 | up | 0.011172302 |
| Rhbdf1 | 2.0266676 | up | 0.011568276 |
| Slc29a1 | 2.3102858 | up | 0.019459581 |
| Gsta1 | 2.1206928 | up | 0.030664629 |
| Ppargc1b | 2.067952 | up | 0.036518283 |
| RGD1311892 | 2.2057195 | up | 0.023151149 |
| Gucy1a3 | 2.3960467 | up | 0.018930428 |
| Ptprd | 2.4984846 | up | 0.010289106 |
| Chga | 2.7510074 | up | 0.024786539 |
| Bcl2l15 | 3.3226822 | up | 0.001146894 |
| Spdef_2 | 2.94326 | up | 0.014196807 |
| Fam195a | 2.2640267 | up | 0.003222098 |
| Ngef | 2.3453372 | up | 0.011280912 |
| Glod5 | 2.2808138 | up | 0.022904529 |
| Ddc | 2.7699373 | up | 0.008394115 |
| Agr3 | 3.0144699 | up | 0.012264248 |
| Tst | 2.7095251 | up | 0.003793983 |
| Degs2 | 2.2255985 | up | 0.002771113 |
| Sult1a1 | 2.8489106 | up | 0.000959741 |
| Kcne3 | 2.2988237 | up | 0.007596873 |
| Bpnt1 | 2.0542249 | up | 0.020652955 |
| Ap1m2 | 2.0151269 | up | 0.029213544 |
| Trim2 | 2.2457324 | up | 0.021993712 |
| Ush1c | 2.1787832 | up | 0.049619537 |
| Gucy2c | 2.6103516 | up | 0.039771918 |
| A_64_P073899 | 2.4682953 | up | 0.019645986 |
| Ms4a12 | 2.02429 | up | 0.009324791 |
| Slc26a3 | 2.0997113 | up | 0.042913618 |
| Slc39a4 | 2.3732093 | up | 0.002408354 |
| Tmem246 | 2.1920851 | up | 0.00788301 |
| Cd99l2_3 | 2.3067772 | up | 0.001132343 |
| Gstk1 | 2.1502231 | up | 0.002282192 |
| Hoxa11 | 3.0548125 | up | 0.001685011 |
| Il17re | 2.0382302 | up | 0.022401416 |
| Me1_2 | 2.4112045 | up | 0.021259251 |
| Nr1h4 | 2.325492 | up | 0.006703096 |
| Nrxn2 | 2.9382942 | up | 0.049580183 |
| Ddah1 | 2.572113 | up | 0.043534157 |
| Ccl11 | 2.642193 | up | 0.045154538 |
| Rgma | 2.3672802 | up | 0.037479058 |
| Klhdc8a | 2.9636676 | up | 0.007581461 |
| Hmcn2_2 | 3.4013843 | up | 0.032879219 |
| Slc22a1_2 | 2.4641015 | up | 0.015830693 |
| Slc25a10 | 2.0862755 | up | 0.009399814 |
| RGD1561381 | 2.5453758 | up | 0.019838501 |
| Chgb | 3.0351567 | up | 0.013850853 |
| Thap4 | 2.1097709 | up | 0.001539778 |
| Prss30 | 2.3108047 | up | 0.00185437 |
| Ugdh | 2.1798212 | up | 0.001181831 |
| Muc2_2 | 3.6791725 | up | 0.027597058 |
| Prdx6 | 3.3429853 | up | 0.040446625 |
| Hmcn2 | 2.7974694 | up | 0.041457206 |
| Aldh1a1 | 2.8294922 | up | 0.047991257 |
| Mab21l2 | 2.6262316 | up | 0.033147971 |
| Slc22a1 | 2.7642958 | up | 0.016307592 |
| Slc4a2 | 2.2447068 | up | 0.015676678 |
| Rgs5 | 4.4718403 | up | 0.009784244 |
| Ptn | 3.8062703 | up | 0.028041875 |
| Slc27a4 | 2.3647107 | up | 0.003314024 |
| Cpt1a | 2.0943923 | up | 0.011689259 |
| Hoxa9l | 2.0148121 | up | 0.034023791 |
| Ethe1 | 2.2088917 | up | 0.020359227 |
| Cryl1 | 2.2768094 | up | 0.005522977 |
| Gsta4 | 2.6949422 | up | 0.001132032 |
| Pck2 | 2.0428421 | up | 0.034819009 |
| Hsd11b2 | 2.0911197 | up | 0.009178409 |
| Akr7a3 | 2.3030722 | up | 0.002355914 |
| Tspan8 | 2.1067155 | up | 0.00206193 |
| Cbs | 2.8939487 | up | 0.002751121 |
| Vil1 | 2.0208086 | up | 0.02463561 |
| Slc12a2 | 2.069508 | up | 0.022306372 |
| Mgat4a | 2.0140919 | up | 0.011003684 |
| Hsd17b2 | 2.7522019 | up | 0.005538577 |
| A_64_P019661 | 3.5398678 | up | 0.034446765 |
| Msn | -2.4190965 | down | 0.042732366 |
| RGD1563145 | -2.3572732 | down | 0.037696908 |
| Rras2 | -2.0692668 | down | 0.001775661 |
| Unc119 | -2.6592151 | down | 0.002483512 |
| Prkcb_2 | -3.3944292 | down | 0.009443739 |
| A_64_P052387 | -2.4460123 | down | 0.039727626 |
| RGD1304595 | -2.1789353 | down | 0.009171169 |
| Il33 | -4.9137235 | down | 0.001523371 |
| A_64_P058689 | -2.3197082 | down | 0.04960188 |
| A_64_P123758 | -2.506667 | down | 0.038350883 |
| A_64_P065363 | -2.1444366 | down | 0.044910587 |
| Mical1 | -2.071026 | down | 0.028627805 |
| A_64_P074953 | -2.3169459 | down | 0.049332359 |
| Nab2 | -2.2653904 | down | 0.010980441 |
| Basp1 | -2.7525333 | down | 0.000584741 |
| Smap2 | -2.2231763 | down | 0.027481325 |
| Lsp1 | -2.8898641 | down | 0.015411458 |
| Ccl21 | -6.4609262 | down | 0.03084857 |
| Mfge8 | -5.3962163 | down | 0.038165607 |
| A_64_P084173 | -2.0586763 | down | 0.040323769 |
| A_64_P157994 | -2.1966847 | down | 0.02298266 |
| Prom1 | 2.2290866 | up | 0.00660366 |
| Noxa1 | 2.0392029 | up | 0.042651581 |
| Chchd10 | 2.2141017 | up | 0.0038612 |
| Mettl7a_2 | 2.158161 | up | 4.09181E-05 |
| Mgst3 | 2.5717546 | up | 0.010231342 |
| Aoc1 | 2.1322548 | up | 0.035417673 |
| Spdef | 2.3796301 | up | 0.045539493 |
| Hmcn2_3 | 2.9291512 | up | 0.025753343 |
| Hmgcs2 | 3.3847005 | up | 0.003346302 |
| Tgfbi | 2.785948 | up | 0.004436106 |
| Sqrdl | 2.5490052 | up | 0.009516623 |
| Atp1a1 | 2.3267873 | up | 0.017453053 |
| Car2 | 3.5219278 | up | 0.001511727 |
| Ca1 | 10.6158746 | up | 0.01912589 |
| Dmbt1_2 | 9.2439065 | up | 0.005237653 |
| LOC290595 | 2.0240094 | up | 0.005071172 |
| Cbr1 | 2.415426 | up | 0.003092973 |
| Selenbp1 | 3.6184872 | up | 0.001778847 |
| A_64_P018633 | 4.3880288 | up | 0.010283899 |
| A_44_P535899 | 2.1935552 | up | 0.041988365 |

The list of genes was generated using a p-value cut-off of 0.05 and a fold-change cut-off value of 2.0 in the transcriptome analysis. In the ‘Direction’ column, ‘up’ indicates genes were upregulated in the S.LEW congenic strain compared to S and ‘down’ indicates genes were downregulated in the S.LEW congenic strain compared to S. P-value was calculated using unpaired t-test.
