# Supplementary material for: Pleiotropic Effect of a High Resolution Mapped Blood Pressure QTL on Tumorigenesis
Source: PLoS One. 2016 Apr 13;11(4):e0153519. doi: 10.1371/journal.pone.0153519 (PMC4830557; doi:10.1371/journal.pone.0153519)
Supplement: S2 Table — The list of genes was generated using a p-value cut-off of 0.005 and a fold-change cut-off value of 2.5 in the transcriptome analysis. In the ‘Direction’ column, ‘up’ indicates genes were upregulated in the S.LEW congenic strain compared to S and ‘down’ indicates genes were downregulated in the S.LEW congenic strain compared to S. P-value was calculated using unpaired t-test. (DOCX) [file pone.0153519.s002.docx]

**S2 Table. List of genes in the heatmap shown in Fig 3B.**

| **Gene Symbol** | **Fold Change** | **Direction** | **P-value** |
| --- | --- | --- | --- |
| LOC100910057 | 3.5903488 | up | 0.003013837 |
| Srd5a1 | 3.6959494 | up | 0.0023343 |
| Trpm4 | 3.4169044 | up | 0.003376761 |
| Ddc | 3.2587301 | up | 0.004377423 |
| LOC100365881 | 2.8378728 | up | 0.000727358 |
| Mettl7a | 2.6472125 | up | 0.003491691 |
| A_64_P138390 | 2.9540229 | up | 0.000247245 |
| Slc4a4 | 3.4385306 | up | 0.001981503 |
| A_64_P163291 | 3.8559036 | up | 0.000244638 |
| Sult1c2a_2 | 4.8959732 | up | 0.002449709 |
| Trpm4_2 | 3.0208635 | up | 0.002350324 |
| Mal2 | 2.8534419 | up | 0.001703338 |
| Ptprd | 2.8267272 | up | 0.004285868 |
| Il33 | -4.9137235 | down | 0.001523371 |
| Unc119 | -2.6592151 | down | 0.002483512 |
| Tmem150c | -2.5081301 | down | 0.000279244 |
| Pde7a | -3.8468 | down | 0.004706498 |
| Akr1b8 | 3.8994572 | up | 0.000361021 |
| Nlrp6 | 3.9451231 | up | 0.000619269 |
| Hoxa11 | 3.0548125 | up | 0.001685011 |
| Pld1 | 2.672576 | up | 0.001720618 |
| Tst | 2.7095251 | up | 0.003793983 |
| Sult1a1 | 2.8489106 | up | 0.000959741 |
| Bcl2l15 | 3.3226822 | up | 0.001146894 |
| Npr1 | 4.384657 | up | 0.001156695 |
| Cbs | 2.8939487 | up | 0.002751121 |
| Gsta4 | 2.6949422 | up | 0.001132032 |
| Hmgcs2 | 3.3847005 | up | 0.003346302 |
| Tgfbi | 2.785948 | up | 0.004436106 |
| Car2 | 3.5219278 | up | 0.001511727 |
| Selenbp1 | 3.6184872 | up | 0.001778847 |
| Basp1 | -2.7525333 | down | 0.000584741 |
| Mettl7b | 4.309276 | up | 0.003915394 |
| A_64_P135449 | 3.5336774 | up | 0.001889041 |
| Cth | 4.7996878 | up | 0.002283302 |
| Sult1c2a | 5.6496838 | up | 0.001728873 |
| Slc6a20 | 2.7617967 | up | 0.000987646 |
| Akr1c13 | 2.661693 | up | 0.002633706 |
| Mgst1 | 2.6523864 | up | 0.002802345 |
| Atp6v0a4 | 5.7090898 | up | 0.004299129 |
| A_64_P148330 | 2.8094514 | up | 0.003940019 |
| Ces2j | 4.1681466 | up | 0.001263659 |
| Ttpa | 3.0529996 | up | 0.001684656 |
| A_64_P006255 | 3.2210609 | up | 0.0004076 |
| Vash2 | 6.4438754 | up | 0.000877682 |
| RGD1559960 | 6.830004 | up | 0.003236271 |
| Smpx | 5.6342002 | up | 0.001891257 |
| A_64_P048705 | -4.5084686 | down | 0.002586488 |
| Msh5 | -5.2548032 | down | 0.002494389 |
| Kcnmb4 | -2.8045503 | down | 0.002598907 |
| Scn4b | -5.4382765 | down | 0.00066085 |
| Mybpc1 | -11.7554536 | down | 0.000293965 |
| Foxs1 | -2.5156862 | down | 0.002983204 |
| A_44_P123818 | 4.8627991 | up | 0.001016516 |
| Bend7 | 3.065226 | up | 0.00115393 |
| Cyp2d2 | 3.6525786 | up | 0.004563305 |
| Wdr72 | 2.7887728 | up | 0.001579095 |
| Cbs_2 | 2.7287104 | up | 0.000412964 |
| Acvr1c | 2.6530769 | up | 0.001881247 |
| Slc26a7 | 6.2250788 | up | 0.000982739 |
| Ntrk2 | 6.0686059 | up | 0.00157409 |
| Zbtb16 | 3.0732895 | up | 0.002284915 |
| LOC680643 | 4.605212 | up | 0.003122024 |
| Slitrk2 | 8.3412911 | up | 0.003657215 |
| Myh1 | -11.1713585 | down | 0.000102875 |
| Sctr | -2.8950302 | down | 0.00278897 |

The list of genes was generated using a p-value cut-off of 0.005 and a fold-change cut-off value of 2.5 in the transcriptome analysis. In the ‘Direction’ column, ‘up’ indicates genes were upregulated in the S.LEW congenic strain compared to S and ‘down’ indicates genes were downregulated in the S.LEW congenic strain compared to S. P-value was calculated using unpaired t-test.
